# Supplementary material for: Prenatal Bisphenol a Exposure, DNA Methylation, and Low Birth Weight: A Pilot Study in Taiwan
Source: Int J Environ Res Public Health. 2021 Jun 7;18(11):6144. doi: 10.3390/ijerph18116144 (PMC8201193; doi:10.3390/ijerph18116144)
Supplement: Supplementary file 1 [file ijerph-18-06144-s001.zip › ijerph-1207603-supplementary.pdf]

Supplementary material

Supplementary Table legends

Table S1 Primer sequences used for pyrosequencing analysis of 8 CpG sites

Table S2 The selection of 87 CpG sites from multiple regression models of BPA exposures, CpG sites and birth weight

Table S3 Causal Mediation Analysis: estimates of natural indirect and direct effects of 2nd ln-transformed BPA (μg/g creatinine) on 8 CpG sites methylation in cord blood and low birth weight

Figure S1 Volcano plots showing $\log_{10}$ (*p*-values) versus the magnitude of effect (partial regression coefficient) of the association between (A) maternal DNA methylation and prenatal exposure to BPA and (B) birth weight and maternal DNA methylation

Table S1 Primer sequences used for pyrosequencing analysis of 8 CpG sites

CG site			5'->3' sequence
cg27640254	1st PCR	1st PCR forward	AGAAAGAAGGGGTAAGGGTGA
		1st PCR reverse	TCAACAAAACCAATCAAATCCCA
	pyro PCR	Uni-F1	GGGACACCGCTGATCGTTTAAATGTTAAGATTTTATATTTTGGGGTAATG
		R1	CACTAACTTAACCCTCAACAAACTTACAAT
	pyro seq	S1	AAAAACCATCCCCCT
cg05524038	1st PCR	1st PCR forward	GTGGAGGGGAGGGTGTTTTA
		1st PCR reverse	ACCTAATACTTCTCACTCACAAACT
	pyro PCR	Uni-F1	GGGACACCGCTGATCGTTTAAAGGTTAGGTTTTGTGGAGTTAGAA
		R1	CCCCAAACAAATCCCTTCA
	pyro seq	S1	ATCCCTTCAACTCTAAAT
cg19768311	1st PCR	1st PCR forward	GGGATTGATGTAGTGTTGTGGT
		1st PCR reverse	CACCTACCAACAAATTAAACAAATCC
	pyro PCR	F1	AGAGGATGTAGATTTATTGTAGGTATAG
		Uni-R1	GGGACACCGCTGATCGTTTATTAATTCTACCAAAACCCTTCCTTAAAC
	pyro seq	S1	AAAATTATATAGATAAAATAAAAAG
cg01502353	1st PCR	1st PCR forward	TGGTGTAATTAGGAAGTTGATTTGGT
		1st PCR reverse	TCCTTTAACTTTATCTTCTATCCCCT
	pyro PCR	F1	GAATTTTATTTTTTGTGGGAAATGAAGG
		Uni-R1	GGGACACCGCTGATCGTTTACCCTCAAATCAAAACCATATATACCC
	pyro seq	S1	AGTTTTATATAGAAATGAAATTAT
cg23244463	1st PCR	1st PCR forward	TGGTATTTTTTGTTGAGAAGGATGA
		1st PCR reverse	ACCTTCCTAACCCACTTCCA
	pyro PCR	F1	GGGAGGTTTGTGTTGTATTGT
		Uni-R1	GGGACACCGCTGATCGTTTAACAACCACATCTATCAATTCCATACTT
	pyro seq	S1	AAAGTAATTAAAAGGAGGG
cg07349217	1st PCR	1st PCR forward	TGTTATTGTGGAGTTTTAAGAGGTTG
		1st PCR reverse	CCACCCCCAAATCCTCAATAAA
	pyro PCR	F1	AGAAGGAGAAAGGGTAGGG
		Uni-R1	GGGACACCGCTGATCGTTTAACCCCATCCTATTTTCTCTACTA
	pyro seq	S1	TTTTTTTAAAGATATAGAGTTTTGG
cg19427642	1st PCR	1st PCR forward	TGTGAGGTTGTGGTTTATAGTATTGT
		1st PCR reverse	TCCCTCTCCTTTCCTTTAACCA
	pyro PCR	F1	ATGGGATTGTTATTTTTGGAGTT
		Uni-R1	GGGACACCGCTGATCGTTTAACTACCAACTATTACTTCCCTTCTTACCA
	pyro seq	S1	GTAGTTAAATATAAAAATATTAGGG
cg27420224	1st PCR	1st PCR forward	GAGGGGTGGGTGAGTTAAGG
		1st PCR reverse	ACACCTACACATTCTCAAATTCCA
	pyro PCR	F1	TGAGTTAGGGTTTTAGTAGTTGTAATTAG
		Uni-R1	GGGACACCGCTGATCGTTTAATACCCTCTCTACCTTCCTTTCAA
	pyro seq	S1	GTATTTAGGGTGTTAGTTAGAA

Table S2. The selection of 87 CpG sites from multiple regression models of BPA exposures, CpG sites and birth weight.

Target ID	Genes	DNA Methylation vs BPA		Birth weight vs DNA Methylation	
		Adjusted ß[1]	p-value	Adjusted ß[2]	p-value
cg23231974	PLOD1*	0.000437	0.02658	-53401.7	0.001093
cg27620176	GRAMD4	0.000449	0.034547	-33028.9	0.040902
cg24891034	ZBTB9	0.000752	0.043202	-25501.9	0.004269
cg13776158	DENND1A	-0.00072	0.023785	-25073.7	0.017005
cg21483415	MUC20	0.000722	0.011955	-24878	0.033755
cg10635194	KCND3*	-0.00077	0.04873	-21128.4	0.015891
cg00762512	RPL9	0.001	0.00669	-20397.6	0.023182
cg17148755	CUX1	-0.00088	0.008611	-20058.7	0.047008
cg18057128	RNF213	0.000857	0.032365	-18409.9	0.030891
cg25411110	HNRNPA3	0.000986	0.006376	-18392.6	0.04699
cg27420224	HNF4A*	0.000963	0.029216	-18292.6	0.016745
cg05577548	SHANK2	-0.00108	0.008577	-17681.9	0.029964
cg05729387	C17orf54	-0.00093	0.036462	-16869.9	0.02835
cg23244463	KCNB2*	0.001079	0.046132	-15590.5	0.012731
cg01370213	LOC283174	-0.00094	0.046349	-15554.6	0.033466
cg12276472	ELL	0.001115	0.047088	-15351.1	0.010666
cg00503852	TM2D1	0.001155	0.049317	-14927.2	0.00926
cg16680506	TGM6	0.001267	0.007906	-14500.2	0.038985
cg27640254	DHRS9*	0.001255	0.021306	-14440.9	0.019362
cg21930229	HDAC1	0.00099	0.036605	-14289.8	0.049764
cg07001909	NRXN3	0.001047	0.033362	-13885	0.047123
cg01975706	JAM2	0.001157	0.036372	-13211.9	0.032989
cg17750640	MGAT4A	0.001292	0.023207	-12842.5	0.031545
cg16701523	SH3BP4	0.001288	0.036177	-12742.9	0.021263
cg24529814	PRDM16*	0.001314	0.018936	-12056.2	0.047712
cg10673246	BTBD11	-0.00165	0.021744	-11573	0.012731
cg24340029	GBGT1	0.001242	0.042952	-11339.9	0.043813
cg06745656	PLXDC1	0.00162	0.007225	-11217.4	0.043517
cg08970083	C3orf70	0.001504	0.04139	-11129.4	0.01549
cg00251716	SDCCAG8*	0.001461	0.035033	-10856	0.027592
cg09171874	S100A3	0.002076	0.03778	-10538.9	0.001156
cg20268341	DNAJB8	-0.00149	0.022236	-10420.2	0.047201
cg01062838	RPL37	0.002194	0.007026	-10317.2	0.010223
cg07832354	PTPRE	0.001484	0.023293	-10294.1	0.049122
cg09580621	CRAMP1L	0.001464	0.049756	-10258.9	0.025623
cg06159352	ELMO1	0.002611	0.006163	-9526.77	0.004829
cg16656078	ELL2	0.00197	0.018173	-9205.08	0.022604
cg11993118	NFATC2	0.001698	0.047059	-8679.4	0.03084
cg18031134	HLA-G*	0.002399	0.033813	-8618.92	0.003058

cg11543686	SLC19A3*	0.001936	0.03024	-8418.36	0.027221
cg24063120	FAM178A	0.001949	0.032745	-7916.05	0.03482
cg05270381	TMEM183A	0.002512	0.024918	-7862.27	0.008133
cg18854735	GNB1*	0.002577	0.023328	-7732.21	0.008201
cg01502353	DST*	-0.00232	0.03181	-7048.97	0.025044
cg24800326	ZNF480	-0.00305	0.003263	-6907.35	0.029041
cg10064322	GIPC1	0.00321	0.021267	-6765.63	0.004041
cg19768311	DST*	-0.00212	0.044918	-6464.35	0.048347
cg08992759	DDN	0.002244	0.047306	-6334.81	0.037659
cg25193867	SYT1	0.003345	0.023973	-6189.42	0.005489
cg16410816	C22orf42	0.00315	0.017709	-6178.91	0.013965
cg01797488	LOC440461	0.002409	0.042717	-6031.91	0.037246
cg21573765	ADCK4	0.003405	0.002165	-5826.94	0.049212
cg09675001	GBP7	0.003342	0.010817	-5629.3	0.027503
cg05386606	BCL7B	0.003288	0.033156	-5443.46	0.012575
cg14525610	SLCO1C1	0.003067	0.048269	-5030.52	0.022347
cg26950369	XIRP2	0.002993	0.039496	-4860.76	0.040111
cg12561776	MYO1D	0.003498	0.036547	-4733.06	0.019775
cg17588350	C11orf64	-0.00336	0.022479	-4705.01	0.042232
cg27535305	SCP2;SCP2	0.003862	0.045947	-4673.97	0.006974
cg22144878	KIAA1012	0.003719	0.04607	-4583.1	0.011469
cg03689601	PUS7L	0.003372	0.038342	-4446.93	0.034916
cg05774672	FZD5	0.004135	0.023989	-4255	0.021045
cg21101631	PCDHGA4	0.004188	0.040686	-4203.05	0.0107
cg05524038	CSF1R*	0.00378	0.032432	-4173.2	0.030858
cg20994022	YBX2	0.00392	0.046387	-4083.34	0.018321
cg11657155	GSTCD	0.003326	0.048797	-4032.08	0.049543
cg20503956	CA13	-0.00414	0.024275	-4013.01	0.030077
cg17527673	SCARF2*	0.003775	0.037227	-3979.99	0.035624
cg00180909	RPS6KA2	0.004065	0.038783	-3972.18	0.0218
cg24127278	CENPA	0.004157	0.049389	-3756.5	0.019995
cg19427642	KCNMA1*	-0.00516	0.017014	-3734.42	0.01566
cg08857906	PPP1R8	0.003989	0.049295	-3383.3	0.047499
cg23845574	VENTX	0.00461	0.044235	-3315.96	0.02651
cg15426035	GAPDHS	0.005425	0.021662	-3265.18	0.022306
cg07349217	TG*	0.005452	0.037903	-3224.26	0.012032
cg18316498	POU2AF1	0.007298	0.00421	-3166.08	0.013138
cg15753394	RBM24	0.005983	0.025833	-3161.28	0.011227
cg01108445	C16orf59	-0.00475	0.039701	-3143.74	0.034416
cg03706175	EPCAM*	0.005094	0.046623	-3095.12	0.020224
cg12135344	CHD5	-0.00497	0.041029	-2895.1	0.041053
cg07577018	ZNF692	0.005759	0.013308	-2894.81	0.046936
cg22943498	BLCAP	-0.00566	0.034429	-2793.64	0.028559

cg00628697	DSCR3	0.00642	0.027318	-2747.67	0.017922
cg09158821	SLC43A2	-0.00657	0.014918	-2618.05	0.036679
cg00636769	GNASAS*	0.007477	0.008173	-2590.99	0.028004
cg07092029	CDH13	0.007616	0.033249	-2529.1	0.006609
cg26939721	SNRPN	0.006387	0.031181	-2299.36	0.04742
cg18380783	DSCR3	0.007173	0.042132	-2046.31	0.035715

* the selected genes in Table2. [1]Maternal DNA methylation=intercept + ß x Ln BPAcr and adjusted for maternal age, pre-pregnancy body mass index (BMI), gestational age (GA), weight gain, infant sex, parity, and pregnancy complications and diseases. [2]Birth weight=intercept + ß x Maternal DNA methylation and adjusted for the aforementioned confounders.

Table S3

Causal Mediation Analysis: estimates of natural indirect and direct effects of 2nd ln-transformed BPA (μg/g creatinine) on 8 CpG sites methylation in cord blood and low birth weight

CpG sites	Gene	Indirect effect (95% CI)	Direct effect (95% CI)	Total effect (95% CI)	Estimated percent mediated
cg01502353 (n=73)	DST	0.001 (-0.002 to 0.005)	0.017 (0.001 to 0.03)	0.018(0.001 to 0.034)	1.5
cg05524038 CpG2nd (n=74)	CSF1R	-6.9e-06 (-2.2e-03 to 2.5e-03)	1.8e-02 (2.6e-03 to 3.3e-02)	1.8e-02 (2.8e-03 to 3.3e-02)	0.05
cg05524038 2CpGs (n=74)	CSF1R	6.9e-05 (-2.9e-03 to 3.5e-03)	1.7e-02 (4.5e-04 to3.2e-02)	1.7e-02 (4.5e-04 to 3.2e-02)	0.14
cg07349217 (n=70)	TG	4.0e-05 (-2.4e-03 to 2.4e-03)	1.8e-02 (2.5e-03 to 3.5e-02)	1.8e-02 (2.1e-03 to 3.5e-02)	0.06
cg19768311 (n=74)	DST	1.4e-05 (-2.79e-03 to2.97e-03)	1.7e-02 (1.6e-03 to 3.2e-02)	1.7e-02 (1.5e-03 to 3.2e-02)	0.06
cg23244463 CpG3rd (n=73)	KCNB2	-0.0002 (-0.004 to 0.004)	0.018 (0.002 to 0.034)	0.018 (0.001 to 0.034)	-0.01
cg23244463 CpG4th (n=74)	KCNB2	-0.0002 (-0.004 to 0.003)	0.017 (0.0003 to 0.033)	0.017(0.0003 to 0.0334)	-0.003
cg27420224 CpG1st (n=72)	HNF4A	-5.1e-05 (-3.0 e-03 to 2.4e-03)	1.8e-02 (1.7e-03 to 3.4e-02)	1.8e-02 (9.2e-04 to 3.4e-02)	-0.07
cg27420224 4CpGs (n=72)	HNF4A	-8.74e-06 (-2.52e-03 to 2.35e-03)	1.74e-02 (8.3e-04 to 3.3e-02)	1.7e-02 (1.0e-03 to 3.3e-02)	0.03
cg27640254 CpG2nd (n=55)	DHRS9	-0.003 (-0.010 to 0.002)	0.026 (0.004 to 0.046)	0.023 (0.001 to 0.044)	-7.2
cg27640254 2CpGs (n=68)	DHRS9	-1.6e-05(-3.1e-03 to 2.4e-03)	2.0e-02 (2.8e-03 to 3.8e-02)	2.0e-02 (2.2e-03 to 3.8e-02)	0.09

cg19427642 site: not available due to small sample sizes in DNA methylation level

(A)

P value
(-log10)

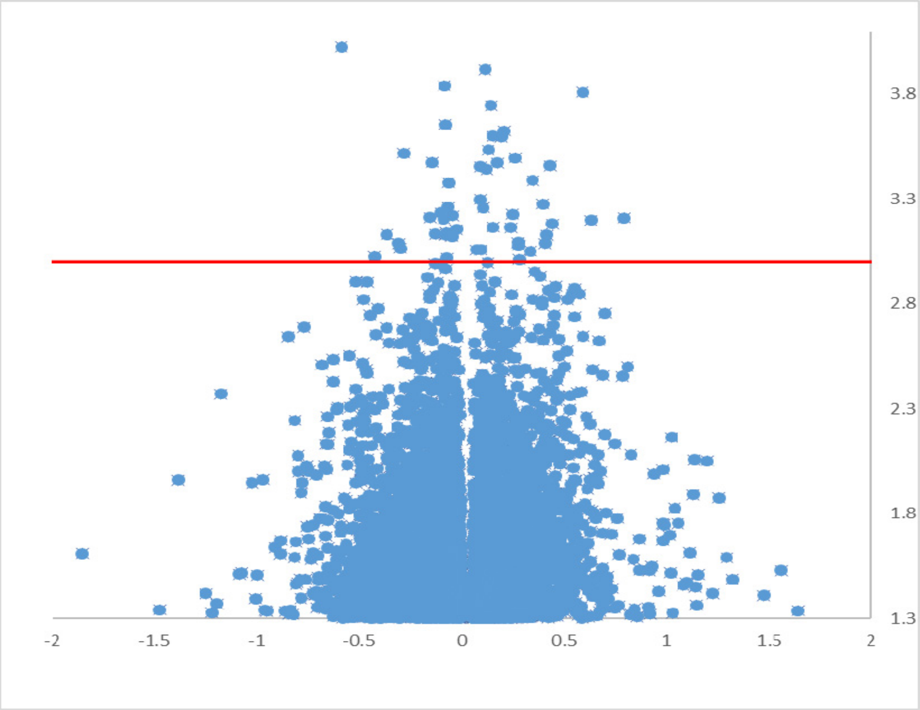

Effect estimate

(B)

P value
(-log10)

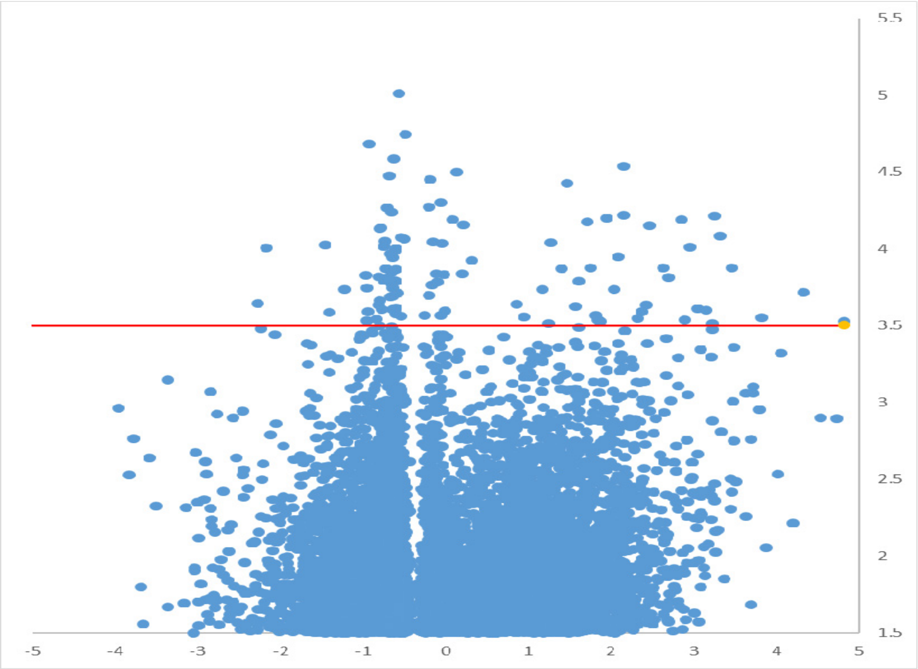

Effect estimate

Figure S1 Volcano plots showing $\log_{10}$ (*p*-values) versus the magnitude of effect (partial regression coefficient) of the association between (A) maternal DNA methylation and prenatal exposure to BPA and (B) birth weight and maternal DNA methylation
